# Six Values Never to Silence: Jewish Perspectives on Nazi Medical Professionalism

**DOI:** 10.5041/RMMJ.10327

**Published:** 2018-01-29

**Authors:** Jacob M. Kolman, Susan M. Miller

**Affiliations:** 1 Houston Methodist Hospital, Center for Outcomes Research, Houston, Texas, USA; 2 Houston Methodist Research Institute, Houston, Texas, USA; 3 Weill Cornell College, New York, New York, USA

**Keywords:** History of medicine, Holocaust, Jewish ethics, medical ethics, medical professionalism, principle-based ethics

## Abstract

An ideological case study based on medical profession norms during the Third Reich will be used to exemplify the importance of diversity in the manifestations of professional ethics. The German professional medical community banned their Jewish colleagues from treating German citizens. This included legally mandated employment discrimination and outright censure which led to a professional ethic devoid of diverse voices. While the escalation to the T-4 program and medicalized genocide was influenced by many causes, the intentional, ethnocentric-based exclusion of voices was an important contributing element to the chronicled degradation of societal mores. For illustration, six core Jewish values—life, peace, justice, mercy, scholarship, and sincerity of intention—will be detailed for their potential to inspire health-care professionals to defend and protect minorities and for readers to think critically about the role of medical professionalism in Third Reich society. The Jewish teachings highlight the inherent professional obligations physicians have toward their patients in contrast to the Third Reich’s corruption of patient-centered professionalism. More fundamentally, juxtaposing Jewish and Nazi teachings exposes the loss of perspective when a profession’s identity spurns diversity. To ensure respect for persons in all vulnerable minorities, the first step is addressing professional inclusion of minority voices.

## INTRODUCTION

With the recent rise of divisive rhetoric in the US and Europe, it is important to remember how ignoring diverse voices can contribute to the ethical deterioration within a culture. For instance, several core Jewish values apply to medicine, including an emphasis on the preservation of life; however, by mid-1933, the professional medical community of Germany banished Jews from their ranks through employment discrimination followed by outright censure, preceding relevant Nazi legal decrees.^1^ Professionals and institutions relinquished their duties to the individual patient in favor of a societal emphasis on economic worth and eugenic hygiene, which, as recent scholarship has shown, even became codified into professional ethics curricula for German medical students.^2^ This obsession with racial-national identity purity escalated into a distinctly medical form of genocide. While the escalation was due to many complex factors beyond the scope of a single paper, the initial ethnocentric-based exclusion of voices from powerful professional institutions contributed to the degradation of social mores. We posit that professional discrimination served not only as a step in the growing persecution of Jews specifically, but also as a means of ignoring cultural teachings which would have resisted professional entanglements in Nazi priorities more generally.

We contrast Jewish teachings with a historical review of Third Reich medicine, in particular the development of the T-4 euthanasia program, as an ideological case study in divergent teachings. This historical example serves to illustrate how the medical profession can fail to serve and *protect* marginalized groups, in part by whether the profession has chosen to include those oppressed voices within itself as a means of self-critique. We will discuss six carefully selected core Jewish values—life, peace, justice, mercy, scholarship, and sincerity of intention—which could have inspired health-care professionals to defend minorities had these core values not been ignored or distorted by the architects of Third Reich medicine. These principles draw on the Jewish canon of Written and Oral Torah, as well as later influential Jewish works addressed specifically to physicians as professionals. Taken together, these teachings repeatedly emphasize the inherent professional responsibility of physicians toward their patients in making decisions about life, health, and ultimately death. These moral errors and ultimate failures of the Third Reich are based on this fundamental disruption of the patient–physician relationship and the corruption of patient-centered medical professionalism.

## HISTORICAL BACKGROUND

Modern-day physicians may not comprehend the extensive role physicians performed during the societal escalation of genocide under the Third Reich. Although the Nuremberg Doctors’ Trial illuminated a subset of the most egregious behaviors,^3^^(pp17–18)^ the medicalization and sustainability of the sterilization and genocidal practices required the systemic support of the medical community. The “why” and “how” of these historical behaviors were based, in part, on professional socialization which converted the physician’s fiduciary responsibility, obligations, and accountability away from patients to the politicized goals of society. Drawing from a broader, existing scholarship on the rise of Third Reich medicine, only a few key events, ideas, and persons will be summarized here as particularly illustrative of the contrasting values discussed below.

The concept of beneficence was applied toward the health of the general population (*Volk*) rather than the individual, gaining prevalence at least two years before Adolf Hitler’s ascension to power; early talk of euthanizing the incurable to prevent a “financial burden” within the economically destabilized post-WWI Germany had begun but was not yet embraced professionally.^2^^(p591)^ After the fall of the Weimar Republic, the scientific theory of eugenics was used to justify the concept of racial-biological hygiene, both legally and ethically. The Nuremberg Law for the Prevention of Progeny of Hereditary Diseases^4^ led to the creation of the Hereditary Health Courts (*Erbgesundheitsgericht*), an infrastructure utilizing physicians and judges to provide scientific justification for decisions which were intended to improve the genetic health and racial purity of the German population.^3^^(p25),^^5^^(pp299–300),^ 6^(pp29–30)^ Practices such as involuntary sterilization and mixed heritage abortions helped ensure racial purity by weeding out undesirable members of the gene pool.^3^^(pp21–29,42)^ The subsequent sterilizations were portrayed as a necessary medical procedure, based on the model compulsory sterilization law proposed by Harry H. Laughlin in the United States.^5^^(p312)^ Beyond legalization, this policy was later reinforced through the Medical Law and Professional Studies (MLPS) medical ethics curriculum, implemented by 1939 and featuring lectures by euthanasia thought-leader Eugen Stähle, and through a key textbook by Rudolf Ramm which redefined the physician’s role as “responsible for ridding society of certain groups ... unable to contribute to society ... in order to heal the organism of the German people.”^2^^(pp593–594)^ Though relatively late in institutional dissemination, this curriculum reinforced what was already professionally accepted. Between 1937 and 1939, concerns were raised that sterilizations had become *over*-applied by zealous physicians caught up in the new industry, and the Nazi party stepped in to systematize the process, which led to the T-4 program, “improving” (among other things) cost efficiency and economic performance.^1^

Although the earlier German sterilization processes resulted in more than 400,000 involuntary sterilizations,^1^,^5^^(p299),^^6^^(p30)^ this program was not considered robust enough to address the perceived threat posed by the unfit. Hence, an escalation of harm progressed to purportedly objective determinations of lives that were considered unworthy of a social safety net and by default, “unworthy of life.”^3^^(p46),cf.^^7^ Within an additional context of rationing war time resources, the Third Reich in August 1939 required the registration of children with defined medical conditions for “special treatment” based on the 1920 writings of Karl Binding, a jurist, and Alfred Hoche, a psychiatrist.^8^^(pp182–188)^ Binding and Hoche argued the law should permit the killing of “incurable ... feebleminded” individuals and suggested the definition of a worthy life was determined by an individual’s social contribution.^3^^(p46)^ Hoche described the economic burdens to society from individuals he described as “human ballast.”^3^^(p47)^ Hoche was a member of Brandt’s board of examiners^9^^(pp33,38)^ and served an instrumental role in teaching Karl Brandt (a key personal physician of Hitler’s)^3^^(pp51,64),^^8^^(p186),^ 9^(p37)^ that “euthanasia” was a “humane” therapeutic goal for the “damaged, useless or harmful.”^9^^(p36)^ Hoche’s teachings “provided the intellectual and moral basis from which Brandt would later argue his case, after Hitler had asked him to implement” the T-4 program.^9^^(p37)^ Both Brandt and Hoche felt “the life of one human being could be sacrificed for the greater good of society or the advancement of medical science,”^9^^(p37)^ thus providing a medical rationale for eugenically based euthanasia programs. The economic burdens of Hitler’s new war also supplemented the earlier (post-WWI) economic justifications, that “the continued existence of those classed defective could no longer be justified in Hitler’s war-strapped Reich.”^5^^(p317)^

Discomfort with T-4, however reasoned away, remained implicit in its lack of transparency and euphemistic means of operation. The central administrative structure for the program was secretly authorized by Hitler in October 1939, and a bureaucratic program was created to camouflage the deaths as natural, and not intentionally caused by the hands of the physicians.^8^^(pp186–191),^^9^^(pp124–129)^ Research was performed to determine the most humane way to shorten individual and group lives, and the lessons learned were later applied in the concentration camps. Euphemisms, such as “merciful act,”^8^^(p188)^ “putting to sleep,”^3^^(p57)^ or “special treatment,”^8^^(p208)^ provided psychological distance for the Nazi physicians to accept these murders as legitimate medical practice and indeed even as a form of mercy. While the bureaucracy diffused individual responsibility, Brandt emphasized that “only doctors were meant to perform the gassing operations,”^9^^(p140)^ echoed also in the motto of T-4 operational head Victor Brack, “the needle belongs in the hand of the physician,”^6^^(p35),^^10^^(p708)^ providing a medical rationale for the next stages of extermination. When faced with opposition, Brandt asserted this professional sovereignty as authoritarian—“doctors could not violate medical ethics, not because they were unable to inflict harm on humans, but because they were doctors. Their professional status freed them from any kind of moral and ethical responsibility towards their patients ...”^9^^(p256)^ As Proctor notes, “Doctors were never *ordered* to murder psychiatric patients and handicapped children. They were *empowered* to do so, and fulfilled their task without protest, often on their own initiative,” and those “who did object, complained primarily that the operation was not, strictly speaking, *legal*.”^8^^(p193)^ A euthanasia law was never formally created beyond the draft stage.^3^^(pp56,64),^^10^^(p706)^

Although Hitler promised the administrators of the T-4 program that “he would bear full responsibility” for physicians’ actions,^8^^(p194)^ both the words of his physician thought-leaders and the relative legal silence regarding T-4 suggest the governmental powers played a secondary role (protecting against liability, first by court rule and then by obfuscating the more extreme activities from public eye) to the central role of the physician, whose values must dictate the destruction of life for economic and racial reasons in order for that destruction to occur.

As other scholars have implied,^1^,^2^^(p593),^^3^^(p40)^ a key facilitator in this drastic professional re-evaluation was isolation from any contrary values. In keeping with the nationalist and racial focus toward the supreme German *Volk*, it was against policy for German doctors to accept the international Nobel Prize (though spreading Nazi medical ideas via hosted conferences was allowed).^3^^(p40)^ The expulsion of Jews from the medical profession proceeded with alarming speed, first locally, then nationally, then by legal decree, all within March and April of 1933.^1^ Through circulation in the prestigious professional publication, *Deutsches Arzteblatt*, and by the directive of Karl Haedenkamp, Jews were first to be replaced so that employment preference could be given to Aryan doctors; they were then barred from practicing on non-Jewish patients; then from treating any insured patients (as a matter of professional enforcement by lobbying with the insurance companies); then legally from such practice as well as from re-licensure.^1^ Propaganda was also utilized, including cartoons and caricatures of Jewish physicians as “anti-healers,” accusing them of rape, abortions, and differential treatment against Aryans, and of treating “sicknesses and not sick people” (the irony and hypocrisy of which is not lost on commentators, who note that Nazis would inflict exactly these wrongs onto the Jewish people).^3^^(pp41–42)^ All such efforts were successful, and “by 1936, all German Jewish physicians were professionally decertified.”^10^^(p707)^ Some exceptional licenses were allowed for the treatment of non-Aryans only, but that still left only an estimated 285 “non-Aryan” physicians in 1938, versus 9,000 “non-Aryan” practitioners in 1933.^1^

It would be a mistake to attribute this forceful exclusion solely to the idea that Jews were convenient targets for Nazis’ racism or nationalism during a period of economic hardship. Physician-educator Rudolf Ramm rejoiced in particular that “the profession had been extensively cleansed of politically unreliable elements foreign to our race.”^2^^(p593)^ A Jewish presence within German medicine would not only be seen as a racial impurity in general, but as a source of “unreliable” ideology contrary to Nazi medical ethics. In order to reinvent the medical profession as one which values individual life by economic and eugenic parameters, discards life for the sake of a monolithic national culture, and furthermore considers doing so to be a justified professional duty, voices had to be silenced first.

## JEWISH VALUES

Six values receive special emphasis within Judaism and have been applied directly to medical practice: *life*, *peace*, *justice*, *mercy*, *scholarship*, and *sincerity of intention*. These values are showcased based on the first author’s own immersive experience learning among multiple Jewish denominations. We do not assert this list as exhaustive, but only as especially prominent and well-attested among the applicable values in Jewish religion and overall culture. These select values will be considered within the structural context of Jewish practical ethics, or *halakhah* from the Hebrew word “to walk” (as in, to walk the ethical path; see Table 1), as well as two direct codes, the Oath of Asaph (a Jewish cousin to the Hippocratic Oath, circa third–seventh century CE)^11^ and the Prayer for Physicians (eighteenth century; though often called the Prayer of Maimonides, and inspired by the twelfth-century rabbi-philosopher-physician, it is notably of much later German origin).^12^,^13^ A glossary of Jewish/Hebrew terms is also provided in Box A.

**Table 1:** Categories of Halakhic Sources in Rabbinic Judaism.

Tier	Sources	Contents
Written Torah	Five Books of Moses	Traditionally parsed to contain 613 *mitzvot* (Commandments)
Oral Torah	Mishnah Babylonian Talmud (TB)* Jerusalem Talmud (TY)	Ancient commentaries and interpretations (Talmud codified 6th century CE), including: Derived rules, Legislated rules, Informal homiletics
	*Responsa*	Later case-based responses extending to the present day
*Minhag* (Local Custom)	*Responsa* Later codifications	Aspects of Judaism that differ by region, arising from Rabbinic authorship or gradual popular adoption
Lexical Codifications	Mishneh Torah (12th century) Shulchan Aruch (16th century)	Influential collections of prior rulings, including *responsa* and *minhag*, organized topically
Progressive Judaism	Alternate responses and commentaries by non-Orthodox movements	More emphasis on individual liberty in decision-making and/or evolving interpretations of canon; traditional *halakhah* may serve in an adaptive or advisory capacity

* Standard Babylonian Talmud citations are given as “TB [tractate] [pg. #] [a/b for page side],” as in “TB Bava Metzia 59b.” The Mishnah and Jerusalem Talmud are divided into tractates, chapter #, and verse # (“Pirkei Avot 1:2” or “TY Pe’ah 1:1”). See www.sefaria.org or www.sacred-texts.org for public domain translations.

Box A **Glossary**

*Aggadah*: Exegetical parables and homilies; non-legalistic (cf. *halakhah*).
*Chayim*: Life, considered sacred in Judaism.
*Chesed*: Mercy; see also *G’milut chasadim*.
*Chillul Hashem*: “Defiling the Name”—refers to blasphemy through actions unbecoming of a Jew, which thus “defile” the reputation of the Jewish God.
*G’milut chasadim*: Acts of loving kindness (from root *chesed* meaning mercy).
*Ger*: Stranger, foreigner, or convert (for nuance, see Table 2).
*Halakhah*: Jewish principles and precedents of ethics and religious law (see also Table 1).
*Kavannah*: Intention or sincerity; often noted in contrast to empty ritual, or as a necessary complement to prevent empty ritual and preserve the emotional or spiritual intent of such actions or prayers.
*Limmud*: Translates as “Learning;” valued as a life-long pursuit in Judaism.
*Lo bashamayim hi*: “It is not in heaven;” a precept of *halakhah* referring to the Talmud story in which miracles fail to overrule the professional standards of a rabbinic majority.^32^
*Minhag*: Local custom (see Table 1).
*Mishnah*: Main canonical text of the Oral Torah, providing a traditional Jewish understanding of the Five Books of Moses, further expounded upon by the Talmud (see Table 1).
*Mishnat chasidim*: Closely translated as “learning of the saints” (or more roughly but contextually appropriate, “expertise of the pious”); refers to moral standards of exemplary persons, stricter than the religious law for the everyday person.
*Mitzvot*: Commandments (see also Table 1).
*Pikuach nefesh*: *Halakhic* principle “to save a life.”
*Responsa*: Rabbinic legal replies (see Table 1).
*Shalom*: Peace; includes connotations of harmony.
*Talmud*: Two canonical commentaries (Jerusalem and Babylonian) on the Mishnah (see Table 1).
*Tikkun olam*: “Fixing the World” through social justice and acts of kindness.
*Torah*: Five Books of Moses (“Written” Torah) and later Talmudic material (“Oral” Torah) (see Table 1).
*Tzedakah*: Charity (for nuance, see Table 2).

### Life (*Chayim*)

Life is sacred in Judaism, as expressed by the *halakhic* principle of *pikuach nefesh* (saving a life)—actions which save a life from danger, or prevent such danger, override other Commandments. *Halakhah* has a legalistic style, allowing nuanced exceptions, case rulings, and circumstantial sub-clauses to carefully qualify what might otherwise be taken as absolute obligations. Obligatory *mitzvot* (Commandments) therefore become sinful (*prohibited* rather than obligatory) if performed contrary to *pikuach nefesh*. Sabbath laws against work *must* be violated to save a life. Fasting on Yom Kippur (Day of Atonement, the holiest of High Holy Days) is *forbidden* if it would seriously endanger health (e.g. for uncontrolled diabetics). Only the most serious prohibitions (idolatry, adultery, and murder) stand in exception to this rule (i.e. religious martyrs are allowed this self-sacrifice since the alternative would have been forced idolatry).^14^,^15^ *Pikuach nefesh* thus prioritizes sanctity of life while also permitting resistance against violent tyrants, even in the face of personal danger.

On the more homiletic side (the less formalistic counterpart of *halakhah*, called *aggadah*), the story of Genesis reinforces this value. Adam is portrayed as the single progenitor of all humanity in order to affirm that the life of each individual human is associated with the inherent worth of all—“anyone who destroys a life ... destroy[s] the world; and anyone who saves a life is as if he saved an entire world.”^16^

Because life is so infinitely holy by these teachings, an ironic quandary for professional medicine arises. One is not supposed to profit from the per-formance of commandments (and protecting life is always commanded), so it would seem that an economically sustainable profession would be banned, as most medical services should be provided as a matter of more general obligation and not for a fee. This can be thought of as a hyper-inflated version of the secular Rule of Rescue, or “the imperative people feel to rescue identifiable individuals facing avoidable death.”^17^^(p2407)^ Being pragmatic, *halakhah* does allow medical practices to charge based on a carefully carved out exception, but the allowance is genuinely *exceptional* in intent and practice—described by one set of commentators as existing only by “a variety of legal manipulations”—and hence heavily hedged in with caveats and regulative restrictions.^18^^(p662)^ Even with the permission to charge reasonable amounts, Jewish doctors cannot turn away patients for inability to pay without committing a sin “almost tantamount to murder.”^18^^(p662)^ In some interpretations, this charge for medical services is not even characterized as direct fee-for-service, but as compensation for the many hours of study required of the physician to make practice possible.^19^

The contrast to the economic rationales of Third Reich medicine speaks for itself. Judaism fears turning away patients unable to pay, whereas T-4 charged the patient’s insurance for involuntary euthanasia, which itself was performed to rid the state of the economic burdens posed by societally defined patient populations.^1^ The German-Jewish philosopher Moses Mendelssohn forecast an opinion on this concept of “public health” in 1842:

“People expendable to the State; useless to the State,” these are statements unworthy of a statesman ... No country can dispense with even the humblest and seemingly most useless of its inhabitants without seriously harming itself. To a wise government not even a pauper is one too many; not even a cripple is altogether useless.^20^^(p175)^

### Peace (*Shalom*)

Shalom can also be translated as wholeness or harmony, and prayers for it constitute the bulk of Jewish liturgy. This value encompasses not only an internal sense of cohesion within and among the Jewish people; it explicitly includes respect for foreigners. Regardless of differences in religious belief or tribe, non-Jews who demonstrate a basic level of morality are considered righteous under a separate covenant with God (defined by seven Noahide Commandments). An act religiously required of a Jew might even *defile* the Name of God (“*chillul Hashem”* in halakhic terms) if its performance would threaten peace between Jewish and righteous Gentile communities. There is a second and third part to the exegesis about Adam cited above, that the Genesis story is also told “to promote peace among the creations, that no man would say to his friend, ‘my ancestors are greater than yours’” as well as to link human diversity to divine grandeur:

A man strikes many coins from the same die, and all the coins are alike. But [God] strikes every man from the die of the First Man, and yet no man is quite like his friend. Therefore, every person must say, “For my sake the world was created” [and we might add, for the sake of every other as well].^16^

Hence the sanctity of life and multicultural peace are not easily separated in Judaism.

The Prayer for Physicians also interweaves *chayim* and *shalom* (life and inclusive peace) by expressing to physicians the value for *all* human life. Its preface praises God for creating the human body “with infinite wisdom,” describing the intricate harmony of “ten thousand times ten thousand organs” working in concert;^12^ disease is likewise portrayed as purposeful, to warn the patient of the dangers to be averted through medical knowledge, rather than as a punishment to be accepted blindly or as a test to be healed through unassisted faith. Learning to identify and combat illness is necessary for patients “to succeed” not only in their healing process, but in life generally.^12^ The prayer makes no distinction between patients based on background, since all human beings experience suffering.^12^ This endorses the opinion of the historical Maimonides, that medicine—particularly through preventive and holistic care—is instrumental to healing, spiritual growth, and worship.^21^ Maimonides treated all patients in his multicultural environment in accommodating terms. For example, in a letter to a Muslim patient, Maimonides quotes the Koran instead of the Torah.^22^ Another patient famously praised Maimonides above the Greeks: “Galen’s art heals only the body, but [Maimonides’] art heals body and soul,” because the patient is valued in body, mind, and soul.^22^^(p550)^

As the Prayer for Physicians and Maimonidean practice in general suggest, Jewish ethics would not support eugenics as a premise for practicing medicine. While it would be consistent with Maimonides to consider cases of medical futility, the reference point for this determination must be the patient’s own good, not the patient’s social “worth” based on eugenic evaluations of ethnic difference. No concern for “public health” or community beneficence has encroached on even the most permissive rabbinic opinions regarding cessation of care or euthanasia.^23^,^24^ In fact, the emphasis is toward restoration of health or a reduction in suffering.

*Shalom* shows up in remarkably subtle and diverse ways in medical *responsa* as well. For instance, Rabbi Jakobowitz argues that observant Jewish patients have no right to refuse physician advice (by *pikuach nefesh* above, to protect life), but he also asserts Jewish doctors should acknowledge the right for Gentiles to refuse treatment.^25^ It is not the doctor’s place to impose religious rules on those of other faiths, not even out of beneficence, as doing so could drive a wedge between communities, and thus be regarded even as blasphemy (*chillul Hashem* noted above, or, in more secular terms, a violation of the public trust in a health-care system that serves their needs). Thus, a physician promotes *shalom* between people by the same route as promoting *shalom* within the person, through a respectful, holistic, and patient-centered approach.

By way of caveat, there is no perfect faith or ideology immune from cultivating in-group biases, and Judaism is no exception, though such ambivalent counterpoints fall far short of the Nazis’ picture of the Jew as anti-healer. The same tribal thinking which entails that a Jewish doctor should not impose Judaism on Gentiles also entails, in several places, that protective measures which apply to Jews would not apply to Gentiles. In particular, rules referring to “your brother” customarily meant only a fellow Jew (e.g. the return of lost persons).^26^ In more extreme cases, commentaries imply Jewish lives are more valuable, as in the Talmudic reading which paradoxically interjects “a life *from Israel*” into the Mishnaic discussion which derived “anyone who saves a life is as if he saved an entire world” from the Adam story.^27^ Disturbing structural parallels come to mind—such as Rudolf Ramm’s emphasis that general ethical precepts in his textbook only apply to Aryan patients,^2^ or the proactive Nazi policies of supporting employment and medical care for Germans and amputee veterans even while initially discriminating against and eventually exterminating Jews.^3^^(p40)^ However, it is notable that both Maimonidean medical ethics and the later German-authored Prayer for Physicians follow the majority readings (in both the Mishnah and Jerusalem Talmud) which lack “from Israel” and instead apply value to life in an unambiguously universal manner. This choice can be explained two ways. One way is descriptive: the Maimonidean version simply follows the more attested trend both in terms of ancient sources,^28^ and in post-Enlightenment Germany—in sermons of universal brotherhood by the early reformer Israel Jacobson^29^ and neo-Orthodoxy founder Samson Raphael Hirsch^20^^(p190)^ (both early nineteenth century). To portray racially supremacist views as a matter of canonical Jewish Law (as Nazis in fact did)^3^^(p41)^ would misread not only the tradition in its full retrospect, but these German-born movements in particular. The second way is normative: an inclusive stance was simply seen as more fitting for a physician to encourage, in order to serve all patients without discrimination. Again in comparison to Ramm and the case of Nazi medical curricula, the shift between inclusion and exclusion may be gradual, but not passive—it was the physician faculty (not solely or even primarily appointed party ideologues) who explicitly chose which norms to endorse and which to condemn when training the next generation of German doctors.^2^

### Justice (*Tzedek*) and Mercy (*Chesed*)

In Judaism, distributive justice (*tzedakah*, righteous giving) and acts of loving kindness (*g’milut chasadim*) are expressed through social action (*tikkun olam*, “fixing the world”), based both conceptually and grammatically on the balance of the two general values, justice and mercy. The Torah and prophetic writings stress the needs of vulnerable populations in particular—the poor, orphans, widows, and “strangers” (Table 2). The Talmud finds so many Torah verses protecting the stranger, in fact, that any mistreatment involves double jeopardy: verbal abuse counts as three sins, plus two for more material harms.^32^

**Table 2:** Difficult Translations.

Term	Concept
*Ger*^30^*	Modern use may suggest a *ger* is a convert to Jewish religion (given contexts of modern nation-states, in which religion, ethnicity, and race have become distinct identity markers). However, the Biblical term refers to any non-Israelite who lived within the Israelite tribal community as an alien. In other words, the intent would cover immigrants and refugees (and arguably ethnic minorities), when translated from the original tribal setting.
*Tzedakah*^31^*	Comparable to the Islamic zakat and related Christian tithes and alms-giving, this type of giving is obligatory, from a root “tz-d-k” meaning “righteousness and justice.” The secular English “charity,” with subtle connotations of voluntariness, virtue, and supererogation, would not be contextually appropriate.

Terse translations of *words* can sometimes obstruct our intention to listen to another culture’s *ideas*. This discussion has two notorious Hebrew-to-English examples as detailed in the table.

* Reference compares Strong’s Concordance and Brown–Driver–Briggs (scholarly Hebrew–English lexicons).

*Tzedakah* can refer to any charitable donation, but in medicine it requires that poor and marginalized groups receive treatment as a matter of social justice, essentially corresponding to modern post-WWII secular principles of justice, universal health-care systems, and global health improvement initiatives.^18^

*G’milut chasadim* are acts of loving kindness which relate directly to caring for the sick on an individual level. This concept is linked to the early origins of Jewish nursing professionals.^33^ Mercy as “loving kindness” is both patient-centered and life-centered when used in the medical context. The Prayer for Physicians states: “May I never see in the patient anything but a fellow creature in pain ... In the sufferer let me see only the human being.”^12^ The ancient physician Asaph exhorts: “do not harden your heart against the poor and the needy; rather have compassion upon them and heal them.”^11^^(p319)^ A doctor’s fiduciary duty is to heal the patient burdened by society, not to heal society burdened by the patient (still less to heal society burdened by a vulnerable population of patients, in violation not only of the value of life but also of justice). This contrasts with Brandt’s misapplication of the concept of mercy in the T-4 program, wherein “the essential question was not whether the programme was ... in itself humane, but whether the method of killing was humane,”^9^^(pp137–188)^ or with Ramm, who had relocated the physician’s mercy towards the body of the German *Volk*, seeing patients as potential pathogens to that reified collective.^2^

### Scholarship (*Limmud*)

Scholarship, as a virtue, is encouraged both culturally and in explicit homilies. For instance, study is described as the greatest Commandment, in the sense that it leads to knowing how to perform all the others.^34^ Intensive study and intellectual curiosity support one’s efforts in leading a moral life, and scientific observation can become a basis for illuminating truth.

Scholarship is obviously relevant to medicine. Explicit codes such as the Prayer of the Physician emphasize life-long learning, intellectual humility regarding the scope of one’s knowledge, and scientific objectivity; in that same spirit, Maimonides practiced critical appraisal of all medical teachings, whether from Jewish, Greek, or Islamic authors.^22^,^35^ His appreciation of diverse sources of knowledge stands in marked contrast to the Nazi ethnocentric science, which banished Jewish scholarship from sight (literally and figuratively).

Objective scholarship requires careful avoidance, or at least identification and management, of conflicts of interest.^36^ Asaph denounces bribery twice: once in reference to doing harm for a bribe and again for becoming an accomplice to sexual misdemeanors,^11^ suggesting that he appreciated conflict of interest not as an abstract idea to be spoken of in generalities, but in terms of the specifically lucrative transgressions and erosions of commitment which deserved direct address. “Do not lust” and “do not shed blood” go without saying to a pious audience, but do not lust *after a patient* or “shed blood by ... dangerous experiment in the exercise of medical skill”^11^^(p319)^ are specifically medical temptations.

According to some interpreters,^19^,^37^,^38^ the Talmud directly addresses physician objectivity with the provocative line, “The best doctors go to hell.”^39^ The line stands in stark contrast to an otherwise pro-medical tradition, so the commentaries read “best” with some nuance. The sort of ideological doctors who seek the “best” for themselves over the patient, or who forget intellectual humility and fail to place their “best” practices under scrutiny, are the more sensible culprits for this verse, rather than physicians who are truly effective at saving lives. On a historical reading, however, “best doctors” likely refers not to biased or compromised doctors but to literal *witch*-doctors, because the Talmud pre-dates any modern distinction between medicine and magic.^40^ Asaph’s Oath (which is contemporary or near-contemporary to the Talmud) also dwells on the illicit use of idols in medicine.^11^ Maimonides enjoyed the emerging proto-science of medieval Islamic medicine and could therefore apply at least some common empirical standards across medical authors of different faiths. Aside from some medieval apologetics against heresy,^40^ Maimonides associated the practice of good, scholarly medicine with careful methodology, whereas bad medicine was marked by superstition or dogmatic metaphysical speculation, better resembling a modern epistemic distinction.

By the Enlightenment context of the Prayer of the Physician, quackery completely replaces sorcery or heresy as the noteworthy intellectual concern. The prayer condemns both political and financial ambitions as “strange thoughts” far removed from wise scholarship and sound practice^12^—a phrase which, though perhaps evocative of the Biblical idea of “strange gods,” limits itself entirely to mundane examples of financial, social, or intellectual pressures to accept the advice of the less knowledgeable instead of responsibly seeking what is right for the patient. The physician must distinguish legitimately wise mentors from “conceited fools,”^12^ though the prayer does understate the challenge of choosing mentors, evidence, and research programs wisely—the modern distinction between science done well and science done poorly (or completely as a “pseudo”-science). Philosophers of science consider this difficulty under a special heading dubbed “the problem of demarcation.”^41^ Historical examples often show a “know it when we see it” basis, but more precise methodological or evidential standards tend, upon philosophical scrutiny, to include something absurd within the definition of proper science (i.e. flat-Earth model), or exclude cases too broadly (e.g. failing to account for epidemiology or germ theory as scientific).

The philosopher’s struggle is no mere theoretical concern, but impacts concrete and life-altering choices facing the professional physician. As already noted above, Brandt chose Hoche with confidence, and eugenics (however flaw-ridden in hindsight) was as promising and mainstream a research paradigm then as genomics potentially is now. The “best doctors” of Nazi Germany studied eugenics and systemically applied it as a social policy, learning their medical ethics from lecturers like Eugen Stähle.^2^ During the Third Reich, physicians did not separate their racial identities, academic ambitions, and ideology from a self-reflective and independent consideration of their generation’s dominant scientific paradigm. These doctors first became morally and intellectually compromised, and, in consequence, murderers, yet contemporaneously speaking—it is disturbing to admit—they followed (what had become) mainstream curricula and research agendas. Any voices that might have spoken contrary to that agenda were already “cleansed” from professional ranks.^2^^(p593)^

It may seem fanciful today to compare the intellectual challenges of medical scholarship to idolatry, as our references to the Talmud and Asaph imply. However, no matter how much the diction may change over time, the result of error remains the same: death. The practice most frequently associated with idolatry in Scripture (whether to Moloch, Baal, or unnamed deities of Canaan) is child sacrifice, typically through fire.^42^ Indeed these paradigm cases of idolatry mark the difference between those faiths respected by Judaism (under *shalom* above) and those condemned—i.e. whose beliefs harm children to protect theoretical effigies. One-and-a-half million children died in the Holocaust for the effigy of Nazi science.^43^ For a more recent medical example, children in the developed world have been placed at risk of measles due to a single case of research misconduct (involving, among other things, conflict of interest on the part of the researcher).^44^ While that was an obvious case redacted relatively quickly, others are not so obvious, and in any event may not be discovered until after review or attempted replication of (already disseminated and popularized) results. Even though modernity has replaced the mystery of discovering true versus false gods with the practice of following true over false evidence, the problem is no less mysterious and the stakes remain high.

In light of such persistent difficulty, professionals must rely on humility to avoid misapplying any knowledge learned (or seemingly learned) through scholarship, recognizing that in the end, good science can be misused just as pseudo-science can mislead. Thus, much depends on the final substantive value of our list: the sincerity and ever-constant mindfulness of a physician’s intention to treat the patient.

### Sincerity of Intention (*Kavannah*)

Typically contrasted to *keva* (routine), *kavannah* refers to mindfulness. Mindfulness attends to the inner meaning behind an outward action, and can be viewed as a vital, indispensable complement to any value expressed through principles or rules. One’s deeds cannot be separated from one’s inner consciousness. Roots of this concept are evident in the Mishnah,^45^ and were revived by the later Hasidism of Eastern Europe,^20^^(pp161–168)^ developed in philosophically sophisticated directions by modern Jewish philosophers like Martin Buber and Emmanuel Levinas.^46^

To these thinkers, even *halakhic* obligations performed in the wrong mindset are meaningless, while tying a shoelace with proper devotion could be holy.^20^^(p163)^ For each Commandment, there is a rightful intention (typically one that imbues ritual *mitzvot* with ethical intent, e.g. using ritual handwashing to meditate upon acts of justice which truly elevate the purity of one’s hands). In medicine, the proper intent is expressed by the cardinal values discussed above, imploring the physician to treat each patient as a person; but what does this mean? To define personhood through a generalized principle would slip out of our search for intention-based *kavannah* by reverting back to formalistic *keva*. The answer is not to *define* people at all, but to enter a *relationship* with them which must frame any other definitional representations (e.g. biological or psychosocial information used to provide medical care). The Nazis *defiled life through definition*, allowing the category “life unworthy of life” to become possible within, rather than anathema to, the culture’s normatively permissible medical thinking. Nazi euphemisms and proceduralism created psychological retreats from any mindfulness that might recognize or resist this definition as the invasive and anti-Hippocratic move that it is, thus allowing physicians to deviate from patient-centered mercy to an amoral justification for ethnic extermination. Rule of law cannot suffice to restrain these retreats—Nazis could legislate around inconveniently protective regulations and even disregard them.^47^ Even values such as life (for whom?), peace (with whom?), or mercy (toward whom?) are vulnerable to misapplication or misappropriation.

Martin Buber portrayed the difference between defining and relating with the objective term “It” and subjective term “Thou.” Clinicians may also see the parallel to the dual medical stances: seeing the patient as a clinical “It” of biological lab results, a diagnostic puzzle to be solved, a research subject/guinea pig, or a pharmacologic equation to be balanced, versus recalling the mortal, vulnerable humanity of each patient as a “Thou.” Both stances are necessary—one to treat the illness, but the other to care for the patient.^48^–^50^

Contemporary philosopher Hilary Putnam attempts to illustrate the depth of this approach for the lay reader, notably invoking the Holocaust itself in his description:

The danger in grounding ethics in the idea that we are all “fundamentally the same” is that *a door is opened for the Holocaust*. One only has to believe that some people are not “really” the same, to destroy all force of such a grounding. Nor is there only the danger of a denial of our common humanity (the Nazis claimed that Jews were vermin in superficially human form!). [*sic*] Every good novelist rubs our noses in the extent of human dissimilarity, and many novels pose the question: “If you really knew what some other people were like, could you feel sympathy with them at all?” But Kantians will point out that Kant saw this too. That is why Kant grounds ethics not in “sympathy” but in our *common* rationality. But then what becomes of our obligations to those whose rationality we can more or less plausibly deny? *These are ethical reasons for refusing to base ethics on either a metaphysical or a psychological “because.*”^46^^(p71)^ (emphasis added)

This approach does not deny the many obvious similarities between members of the moral community (or between those who *ought* to be considered equal members of the community). Rather it denies that building a *theory* on that similarity would ever provide a foolproof grounding for ethics. Putnam’s choice of Kant (instead of, say, utilitarianism, with its known problems of including respect and justice among its values)^51^ illustrates the challenge of capturing good intention through theory. Ostensibly, Kant speaks strongly of *human* dignity, but his criteria have been criticized for being thoroughly ableist (basing moral dignity entirely in rationality and thereby failing to situate the cognitively impaired within the moral community).^52^–^54^ Thus the Nazis did (“more or less plausibly” from a Kantian perspective) deny rationality and thereby personhood to the disabled and (*implausibly* but nevertheless effectively) to ethnic and political targets. From the stance of Buber and Levinas, there are no rules to abuse or to dodge by crafting exceptions—the reality of another person is basic, preceding all understanding. As long as we believe we can totally understand the Other, we can become confident enough to undermine the Other.

With *kavannah*, one must sincerely face the interpersonal impact of following (or disobeying) laws, engaging in research, or accepting career incentives, with the presence of the Other’s eyes always in mind. Unlike the other five values offered above, philosophies which develop this value offer no guidance or code for the medical professional, only an experience: that a moment of interpersonal responsibility situates and transforms all other tasks (no matter how mundane). Because relational ethics is taken as primary, the Self–Other relation in Levinas and the I–Thou relation in Buber have been widely used to meditate on professional–patient interactions, including hospice care,^55^ gerontology,^50^ and mental health,^48^ addressed to and by physicians and their medical students,^49^,^56^–^58^ nurses,^59^ and spiritual care professionals.^60^ Moments of interpersonal response beyond any expressible principle (other than *hineni*, or “I am here,” the term Abraham uses to respond to God)^56^ often are (and need to be) a shared experience among clinical professionals.

## VALUES AND PROFESSIONAL ACCOUNTABILITY

Professionals operate within (and must relate their values to) a framework of expertise, authority, community role, culture, and occupational code. These criteria distinguish professions (medical and otherwise) from other work.^61^ Incorporating the six values above might help guide a physician’s actions, particularly thanks to the specific *responsa* and physician codes referenced throughout, but our discussion would be incomplete without examining how professionalism itself frames the six values because of this added context; different accounts of professional responsibility can lead to radically different ways a professional may be expected to implement any set of core values. When the values held by the professional, the public, and the political infrastructure diverge, questions of professional autonomy versus accountability arise, which necessarily frame any values adopted.

For instance, Brandt and Brack considered the T-4 program to be the specific and exceptional domain of physicians, a secret domain in which physicians could not be held externally accountable except to the highest level of government (which for the Third Reich was synonymous neither with public scrutiny nor with legal consistency, but with the equally unaccountable fiat of a dictator). In contrast to an unchecked principle of authoritative autonomy, Judaism has two principles of *halakhah* in response*.*

*Lo bashamayim hi* (“the teaching is not in heaven”) is based on the following story in which two sages refuse to change their official ruling in a case of *halakhah*, even when miracles and Heavenly voices are conjured against them by a third sage:

... After failing to convince the Rabbis logically, Rabbi Eliezer said to them: If the *halakha* is in accordance with my opinion, this carob tree will prove it. The carob tree was uprooted from its place one hundred cubits, and some say four hundred cubits. The Rabbis said to him: One does not cite *halakhic* proof from the carob tree.

[Two additional miracles follow, neither of which the rabbis accept as valid proof.]

Rabbi Eliezer then said to them: If the *halakha* is in accordance with my opinion, Heaven will prove it. A Divine Voice emerged from Heaven and said: Why are you differing with Rabbi Eliezer, as the *halakha* is in accordance with his opinion in every place that he expresses an opinion?

Rabbi Yehoshua stood on his feet and said ... “It is not in heaven” (Deuteronomy 30:12). ... Rabbi Yirmeya [explains this passage]: Since the Torah was already given at Mount Sinai, we do not regard a Divine Voice, as You already wrote at Mount Sinai, in the Torah: “After a majority to incline” (Exodus 23:2). Since the majority of Rabbis disagreed with Rabbi Eliezer’s opinion, the *halakha* is not ruled in accordance with his opinion. ... Years after, Rabbi Natan encountered Elijah the prophet and said to him: What did the Holy One, Blessed be He, do at that time, when Rabbi Yehoshua issued his declaration? Elijah said to him: The Holy One, Blessed be He, smiled and said: My children have triumphed over Me; My children have triumphed over Me.^32^

The subject of the sages’ original argument is rather insignificant (it regards the kosher status of a particular sort of oven). The real story implies that wisdom is accessible to human deliberation and that professionals should eschew uncritical obedience to authority merely for authority’s sake; consistency with reason and procedural justice matter as well. This alternative concept of professional autonomy would already insulate a professional’s judgment from the sway of a dictator, but not necessarily for a willing collaborator (or even an intellectual contributor) to Hitler’s mission, such as Brandt.

For that, *mishnat chasidim* (“expertise of the pious”) offers a necessary adjunct principle to professional autonomy: that of a value-laden sense of *responsibility*. *Mishnat chasidim* describes prophets, sages, and legendary rabbis who have enhanced accountability, and how the consequences of their failure affect the entire community. Excellence does not mean that great men are great because they exceed their obligations, but that “a great man has higher obligations than other people” at baseline, precisely because he is great.^62^^(p203)^

One story recounts a Talmudic sage whose clay vessels were broken by day laborers. It is normally within the law to charge workers for damage by negligence, but in this case the sage was required to pay them normal wages and take the damages himself, having the greater privilege and wisdom. *Mishnat chasidim* also applies to utilitarian dilemma cases (e.g. whether to give up a fugitive seeking sanctuary if the authority threatens violence to the community for non-compliance; whether to split scarce water that could save one life to provide insufficient hydration for many lives). In each of these cases, the common person’s answer is the one which maximizes benefits or adheres to a stringent concept of justice (charge the workers; give up the fugitive; hoard the water), while the sage’s obligation leans towards “mercy and self-renunciation,” even when such unyielding principles lead to worse outcomes.^62^^(p200)^

Even professional ethics have limits, however. Dilemmas can make any decision impossible to justify. After studying cases of Jewish prisoner-physicians in concentration camps, Tessa Chelouche finds several instances in which the physician was forced into “unethical” behavior, including lying, stealing, falsifying patient records, and abortions.^10^ Ironically, some of these examples are actually defensible from a *halakhic* perspective, as “the usual code among prisoner doctors was that they would try to do everything to save lives,”^10^^(p710)^ and compared to that, other obligations are forfeit (by *pikuach nefesh*); abortion is a complex case but arguably also falls under *halakhic* precedent to favor the life of the mother over unborn life.^63^^(pp36–38,129–131)^ Other cases were impossible to adjudicate by any rule or principle, as not even *pikuach nefesh* allows the sacrifice of one life for another. These cases included rationing life-saving drugs, infanticide (both to spare children the gas chamber and to avoid adult casualties, whom the Nazis would put to death for procreating), euthanasia, and collaboration in inhumane experiments (and in the system in general). Choosing the path of *mishnat chasadim*, allowing worse outcomes for the sake of principle as the Talmudic case studies suggest, and even martyring oneself rather than collaborating, would also condemn the doctor’s patients to worse fates. Thus, *in extremis*, the real difference between professionals’ behavior is still *kavannah* – as Chelouche’s assessment similarly implies:

The juxtaposition of the Nazis’ use of medicine to inflict pain and suffering on innocent victims with the Jewish doctors’ attempts, in the absence of even the most basic tools, to alleviate suffering and preserve life demonstrates the diametrically opposed purposes to which medical skills could be put.^10^^(p715)^

The cost of true professional intention is also high. Whether ethical principles were available as rationales or not, they would bring the prisoner-physicians little comfort. Their decisions (including the decision to recount them to historians later) are described as “tortured ... excruciatingly painful,”^10^^(p714)^ and that “once [they] realized that the decision of life and death was in their hands, the responsibility crushed them. They had to justify their actions before their consciences.”^10^^(p711)^ Doctors in Nazi Germany’s academia were being taught, in the words of Eugen Stähle, “The fifth commandment ‘you shall not kill’ is not a commandment of God but a Jewish fiction.”^2^^(p593)^ The prison doctors, conversely, did what was necessary in the system, without fooling themselves into thinking the system itself was right, or even that what was *necessary* was right. In the example of abortion, one analyst puts *halakhic* decisions in context: “... the landscape of the Holocaust bore no parallel to any other experience, such that rabbinic rulings from within that world cannot be seen, in any way, as creating precedents for *halakhic* rulings in ‘normal’ times.”^63^^(p129)^ Rather than allow a theoretical basis of ethics (“a because,” as Putnam calls it) to normalize an atmosphere of death, the prison doctors considered only the lives in front of them, as best as they could.

## CONCLUSION

Even for individuals who are non-observant followers of specific faith traditions, the ethical values presented here can provide guidance in modern-day decisions. By exploring the bioethical premises and motivations of Jewish teachings, a physician can recognize (1) life is truly sacred, and neither naturally occurring genetics nor socially occurring diversity in culture should be discounted as “inferior” forms of life (*chayim*); (2) co-existence should be strived for (*shalom*); and (3) social justice and compassion (*tzedek v’chesed*) entail accommodating the sufferer as sufferer in spite of disability. Whether based on eugenics, genomics, or transactional economics, any scientific program (*limmud*) which can suggest the violation of these principles must face constraints from professional ethics. The *locus* of responsibility for this determination is on the physician as professional, and the *focus* can only ever be on the patient. This emphasis bars any Brandt-like notions of “mercy” toward the “unworthy.” Totalitarian medicine was able to operate outside of these values, in part, by silencing the voices that might say “this is not right,” and then normalizing the behavior of killing behind false intentions (a failure of *kavannah*). Nazi doctors considered (and trained their students to consider) their behaviors ethical, but political ideology and incentives which emphasize the interests of society over caring for individuals dislodge the righteous foundations of medicine, and such dislocations should be resisted whenever and wherever they occur.

As society continues to explore the complex politics of health-care systems, so too its relevant professionals must take part in a self-reflective and diversified professional community to minimize moral errors. Complacency about even the slightest in-house discriminatory practice(s) or barrier(s) to representation hampers a profession’s ability to engage in such reflection. Our current world tests moral intuitions on an increasing number of issues: the rise and renewed popularization of genetic testing, privacy in a digital age of big data research, learning health-care systems which blur the boundaries between research and practice, end-of-life care economics, access to reproduction options, gender and identity politics, RVU-based (relative value unit) compensation, and disparity-based access to health care, to name a few. Before crafting advocacy responses to these issues, professionals must consider whom they represent and for whom they are responding, in terms of individual and societal motivators.
